# Does the inflammatory potential of diet affect disease activity in patients with inflammatory bowel disease?

**DOI:** 10.1186/s12937-019-0492-9

**Published:** 2019-11-04

**Authors:** Parvin Mirmiran, Nazanin Moslehi, Nava Morshedzadeh, Nitin Shivappa, James R. Hébert, Farnaz Farsi, Naser Ebrahimi Daryani

**Affiliations:** 1 Nutrition and Endocrine Research Center, Research Institute for Endocrine Sciences, Shahid Beheshti University of Medical Sciences, Tehran, Iran; 2 Department of Clinical Nutrition and Dietetics, Faculty of Nutrition and Food Technology, National Nutrition and Food Technology Research Institute, Shahid Beheshti University of Medical Sciences, Tehran, Iran; 3 Student Research Committee, Department of Clinical Nutrition and Dietetics, Faculty of Nutrition and Food Technology, National Nutrition and Food Technology Research Institute, Shahid Beheshti University of Medical Sciences, Tehran, Iran; 4 Cancer Prevention and Control Program, University of South Carolina, Columbia, SC USA; 5 Department of Epidemiology and Biostatistics, Arnold School of Public Health, University of South Carolina, Columbia, SC USA; 6 Colorectal Research Center, Iran University of Medical Sciences, Tehran, Iran; 7 Department of Gastroenterology, Imam Khomeini Hospital, Tehran University of Medical Sciences, Tehran, Iran

**Keywords:** Dietary inflammatory index, Empirically derived inflammatory pattern, Inflammatory bowel disease, Disease activity

## Abstract

**Background:**

Diet is an important modulator of inflammation, which is associated with inflammatory bowel disease (IBD). In this study, we examined whether the inflammatory properties of diets are associated with disease activity in patients with IBD.

**Methods:**

A cross-sectional study was conducted on 143 IBD patients, including 32 patients with Crohn’s disease (CD) and 111 patients with ulcerative colitis (UC). Dietary intakes were assessed by a valid 168-item food frequency questionnaire (FFQ). The inflammatory potential of the diet was assessed by calculating the two scores of Dietary Inflammatory Index (DII®), and the Empirical Dietary Inflammatory Pattern (EDIP), and CD and UC disease activity were determined by the Crohn’s disease activity index (CDAI) and the Mayo score, respectively. Associations of the inflammatory indices as median and as tertiles with disease activity were analyzed using logistic regression in a univariate model and after adjusting for total energy intake (continuous), type of disease (CD and UC) and drug consumption (no drugs, single drug, and multiple drugs).

**Results:**

Sixty-four IBD patients (44.8%) in this study had active disease.The DII® score and the EDIP did not differ significantly between active and inactive patients (− 1.45 ± 1.04 vs.− 1.20 ± 1.24; 0.56 ± 0.22 vs. 0.53 ± 0.28, respectively). After adjusting for energy intake, drug use, and IBD type, the odds (95%CIs) of active disease among patients in tertile 3 compared to those in tertile 1 were 0.84 (0.32–2.17) for DII and 1.50 (0.61–3.72) for EDIP; neither of which were statistically significantly different from the rates in tertile 1.

**Conclusions:**

Although point estimates were in the expected direction of increased risk, the inflammatory potential of diet, assessed using DII or EDIP, was not associated with severity of disease in IBD patients. Whether diet-related inflammation affects disease activity in patients with IBD deserves further investigations.

## Background

Inflammatory bowel disease (IBD), characterized by chronic or relapsing inflammation of the gastrointestinal (GI) tract [1], is thought to be a relatively common enteropathy worldwide with the highest annual incidence rate of 24.3 per 100,000 person-years for ulcerative colitis (UC) in Europe and the lowest incidence rate of 5 per 100,000 person-years for Crohn’s disease (CD) in Asia and the Middle East [2]. An increasing burden of IBD in Iran suggests that the disease could become an important public health concern in forthcoming years [3].

The etiology of IBD remains unclear but a combination of environmental, genetic, and immune-regulatory factors are thought to be involved [4]. Although the two principal types of IBD can be differentiated by the location of diseases in the gastrointestinal tract, clinical and histological manifestations, complications and prevalence, both types are caused by chronic intestinal inflammation [5].

Food components can affect incidence of IBD and its development at least through modulation of intestinal microbiota, intestinal immune system and inflammation [6]. Studies investigating dietary determinants in the onset of IBD are increasing and some of these suggest meat [7], dairy [8], fiber [9], and vitamin D [10] may influence the risk of developing IBD. Among dietary components, increased attention is being focused on the intake of fish and n-3 fatty acids in the etiology [11] and maintenance of remission of IBD [12] although these studies provided inconsistent findings. Patients with IBD believe that certain foods affect their disease activity [13]. Contrary to the common belief, except for n-3 and fish oil supplementation, the association of other dietary components with disease activity or relapse in IBD patients has not been well investigated [14]. The overall inflammatory properties of the diet may influence disease activity in the inflammatory disease. To assess the properties, dietary inflammatory indices have been developed [15, 16]. The Dietary Inflammatory Index (DII®) has been created based on the inflammatory scores of 45 food parameters which are mainly nutrients. Inflammatory properties of the food parameters were obtained from studies published on association between diet and six inflammatory markers through 2010 [15]. The score has been significantly associated with inflammatory markers [17, 18] and increased risk of diseases characterized by inflammation [19, 20]. The Empirically Derived Inflammatory Pattern (EDIP) has also been proposed to calculate inflammatory dietary score, based on intakes of food groups. The weighted sum of the 18 food groups is used to calculate EDIP scores. Inflammatory properties of the food groups were determined using reduced rank regression considering CRP, IL-6, and TNF-α as response variables, after which their weights were derived using stepwise linear regressions [16]. The ability of the score to predict inflammatory markers of CRP, IL-6, TNF-α, and adiponectin has been shown [21]. A higher EDIP score was associated with higher risk of colorectal cancer [22] and metabolic unhealthy phenotype in overweight/obese adults [23]. Results of a study that examined the association between the DII and risk of UC, suggests an increased risk with higher adherence to the pro-inflammatory diet [24]. To date, no study has investigated the association of the dietary inflammatory indices and the clinical course of IBD patients. Therefore, the aim of this study was to investigate the associations of inflammatory properties of diet, using the DII and EDIP scores, with disease activity in IBD patients.

## Method

### Study population

A cross-sectional study was performed on 143 IBD patients including 32 with CD and 111 with UC*.* In an effort to reduce the possibility of changes in dietary intake adjustment following IBD diagnosis, only newly diagnosed patients with IBD < 6 months were selected from among adult IBD patients, aged ≥18 years, referred to Gastroenterology Clinic of Rasool Akram Hospital in Tehran during 2017–2018. The diagnoses of CD and UC were made based on physical, colonoscopic, and histological examinations. Those patients with history of gastrointestinal surgery, other gastrointestinal illnesses, carcinoma, diabetes mellitus, and hyperthyroidism were excluded. In addition, those on special diets (e.g. vegan, Atkins), and pregnant and breastfeeding women were also excluded from the study; CD and UC disease activity was determined by the Crohn’s disease activity index (CDAI) and Mayo score, respectively [25, 26]. Active disease was defined as CDAI ≥150 and Mayo score < 2 for CD and UC patients, respectively. Ethical committee approval was obtained from the Student Research Committee of Shahid Beheshti University of Medical Sciences.

### Anthropometric and demographic parameters

Data on age, gender, smoking, education, drug consumption, and past medical history were collected using a demographic and medical history questionnaire. Participants were categorized based on education into three groups: < 14 years of formal education, bachelor’s degree, master’s degree and higher. Drug consumption was categorized into three groups: No drug, a single drug (consumption of aminosalicylic acid compounds (5-ASA) or immune modulators, i.e. azathioprine, prednisolone and methotrexate, each per se, and multiple dugs (combination at least two classes of drugs, including ASA, immune modulator, and anti-tumor necrosis factor (TNF) agents, i.e., Remicade® infliximab and Humira® (adalimumab).

Weight was measured using Seca scales (Germany) to the nearest 0.1 kg (without shoes and with light clothing) and height was measured to the nearest 1 cm without shoes. Body mass index (BMI) was calculated as weight in kilograms divided by height in meters squared.

### Dietary assessment and DII® and EDIP calculation

Nutrition information of the patients during the preceding year was assessed using a 168-item food-frequency questionnaire (FFQ) [27]. Frequency of food intake and the amount consumed were recorded and converted to grams. Nutrient composition of the Iranian foods and United States Department of Agriculture (USDA) food composition table were used to determine nutrients intakes. The validity of the FFQ was assessed among a subsample of 132 cohort participants against 12 daily dietary recalls collected over a year. Using the FFQ and 12 daily dietary intakes, acceptable correlations were found between nutrients intakes; ranging from 0.24 for vitamin A to 0.70 for thiamin in men and from 0.11 for beta-carotene to 0.60 for fiber in women [27]. Assessing reliability of the FFQ by completing the FFQ twice with an interval of 14 month also showed good correlations ranged from 0.41 for mono-unsaturated fat to 0.79 for protein in men, and from 0.39 for mono-unsaturated fat and 0.74 for saturated fat in women [27].

Inflammatory potential of diet was assessed by calculating the DII**®** and EDIP scores [15, 16]. To calculate the DII, dietary intakes of 38 food parameters, including energy, carbohydrate, protein, total fat, cholesterol, saturated fat, trans fat, mono-unsaturated fat, poly-unsaturated fat, n-3 fatty acid, n-6 fatty acids, fiber, thiamin, riboflavin, niacin, folic acid, vitamin B6, vitamin B12, vitamin A, vitamin C, vitamin D, vitamin E, iron, selenium, magnesium, zinc, beta-carotene, flavan-3-ol, flavones, flavonols, flavonones, anthocyanidins, isoflavones, caffeine, pepper, tea, garlic, and onions were used; to do this, each food parameter was first standardized using the global means and standard deviations (SDs) values; standardized values were then converted to centered percentiles and then multiplied by the overall food parameter specific inflammatory effect scores. The food parameter specific inflammatory scores of each food parameter were summed up to obtain the overall DII score. To control for the effect of total energy intake, DII per 1000 kcal was calculated.

According to the Tabung et al. study, dietary intakes of 15 food groups including tea, coffee, dark yellow vegetables, leafy green vegetables, snacks, fruit juice, pizza, processed meat, red meat, organ meat, other fish, other vegetables, refined grains, high-energy beverages, and tomatoes were used to develop the EDIP. Food items included in each food group are presented in Table 1. First, mean daily intake of each food group was divided by a group specific portion size [28] to determine its intakes based on serving sizes; these values were then multiplied by their specific inflammatory coefficients [16] and summed up and the final values were rescaled by dividing by 1000 to decrease the magnitude of the score and to simplify the interpretation. The greater scores (more positive) indicate more pro-inflammatory diets and the lower scores (more negative) indicate more anti-inflammatory diet.

**Table 1:** Food groups used to calculate the Empirical Dietary Inflammatory Pattern (EDIP) score

Food groups	Food items
Pro-inflammatory	
Processed meat	Sausages, salami
Red meat	Beef, lamb, hamburger patty
Organ meat	Beef / chicken liver, other organ meats of lamb and beef
Other fish	Canned tuna, fish other than dark-meat fish
Other vegetables	Celery, mushroom, green pepper, corn, eggplant, zucchini, cucumber
Refined grains	White bread, muffin, bagel or roll breads, white rice, pasta, biscuits, noodle
High energy beverages	Soft drinks, other carbonated beverages with sugar or fruit punch drinks
Tomatoes	Fresh Tomato, tomato sauce
Anti-inflammatory	
Tea	Dark tea
Coffee	Coffee
Dark yellow vegetables	Carrots, winter squash
Leafy green vegetables	Spinach, lettuce, mixed vegetables
Snacks	Potato chips, corn chips, pufak (extruded corn snack), popcorn, cracker
Fruit Juice	Apple, orange, cantaloupe juices
Pizza	Pizza

### Statistical analysis

Characteristics of participants were determined based on disease activity. Comparisons between active and inactive disease were made using Student’s – t test for continuous variables and Chi-square for categorical variables. Participant characteristics across tertiles of the inflammatory indices were also compared using ANOVA test for continuous variables and Chi-square for categorical variables. Inflammatory indices were analyzed as categorical variables using both median cut-point and tertile cut-points. Associations between the inflammatory indices and disease activity were analyzed using logistic regression in a univariate model and after adjusting for total energy intake (continuous), type of disease (CD and UC) and drug consumption (no drugs, single drug, and multiple drugs). In this study, age, sex, smoking, BMI, education, drug consumption, type of IBD (UC/CD), and energy intakes were selected as potential covariates, and those with significant univariate association with the risk of active disease (*P* < 0.1) were included in the adjusted model. For statistical analyses, SPSS**®** 20 (IBM Corp, Armonk, NY) was used and *P* < 0.05 was considered significant.

## Results

Of 143 participants recruited in this study, 64 individuals (44.8%) had active disease, of whom 43 (67.2%) had UC. Characteristics of participants based on activity state of disease are presented in Table 2. Age, sex, BMI, number of smokers, and education were not significantly different between patients with inactive and active diseases. There were significant differences in drug consumption between patients with inactive and active disease, with consumption of multiple drugs being significantly higher in active (*n* = 52 (81.2%) vs. inactive patients (*n* = 43 (54.5%), *P* = 0.001). Intakes of energy, macronutrients, and food groups did not differ significantly according to activity of disease. Median and range of DII**®** and EDIP in all participants were − 1.57 (− 3.66, 1.99) and 0.54 (− 0.25, 1.31), respectively. Inflammatory diet scores of DII and EDIP did not differ significantly between the two groups.

**Table 2:** Characteristics of participants based on disease activity ^a^

	Total (*n* = 143)	Inactive (*n* = 79)	Active (*n* = 64)	*P* value
Age, years	38.7 ± 10.8	37.9 ± 11.2	39.7 ± 10.3	0.32
Female, n (%)	66 (46.2)	38 (48.1)	28 (43.8)	0.62
BMI, kg/m^2^	25.0 ± 3.03	25.3 ± 3.21	24.6 ± 2.78	0.19
Smokers, n (%)	22 (15.4)	11 (13.9)	11 (17.2)	0.64
Education, n (%)				
< 14 years of formal education	26 (18.2)	18 (22.8)	8 (12.5)	0.21
Bachelor’s degree	73 (51.0)	36 (45.6)	37 (57.8)	
≥ Master’s degree	44 (30.8)	25 (31.6)	19 (29.7)	
IBD Type, n (%)				
Crohn’s disease	32 (22.4)	11 (13.9)	21 (32.8)	0.006
Ulcerative colitis	111 (77.6)	68 (86.1)	43 (67.2)	
Disease activity, Median (min, max)				
Crohn’s disease activity index	193 (65.0,400)	80.0(65.0, 145)	250 (155, 400)	
Mayo score for Ulcerative colitis	1.0 (0, 11.0)	1.0 (0, 2.0)	8.0 (4.0, 11.0)	
IBD-related drug consumption, n (%)				
No drugs	6 (4.20)	3 (3.80)	3 (4.70)	0.001
Single drugs^b^	42 (29.4)	33 (41.8)	9 (14.1)	
Multiple drugs^c^	95 (66.4)	43 (54.5)	52 (81.2)	
Dietary intakes				
Energy (kcal/d)	2021 ± 326	2062 ± 354	1971 ± 282	0.09
Carbohydrate (% of energy)	62.4 ± 4.16	62.2 ± 4.77	62.8 ± 3.26	0.40
Fat (% of energy)	26.2 ± 3.60	26.4 ± 4.10	26.0 ± 2.89	0.51
Protein (% of energy)	14.2 ± 1.38	14.3 ± 1.56	14.1 ± 1.11	0.46
Saturated fatty acids (g/d)	17.6 ± 4.57	18.3 ± 4.94	17.0 ± 3.95	0.06
Monounsaturated fatty acids (g/d)	21.5 ± 4.61	22.0 ± 5.32	21.0 ± 3.5	0.23
Polyunsaturated fatty acids (g/d)	12.7 ± 2.61	12.8 ± 2.95	12.6 ± 2.11	0.65
Grains (g/d)	497 ± 167	505 ± 200	487 ± 112	0.52
Legumes (g/d)	13.2 ± 14.0	13.2 ± 14.7	13.3 ± 13.2	0.96
Meats (g/d)	64.2 ± 28.8	66.9 ± 33.8	60.8 ± 20.9	0.21
Dairy products (g/d)	233 ± 155	251 ± 162	297 ± 89.3	0.12
Fruits (g/d)	293 ± 78.1	297 ± 89.3	288 ± 61.7	0.51
Vegetables (g/d)	191 ± 48.7	190 ± 53.7	193 ± 42.1	0.72
Fast foods (g/d)	13.9 ± 15.1	14.6 ± 18.4	13.0 ± 9.67	0.53
Diet inflammatory scores				
DII	−1.59 ± 1.24	−1.21 ± 1.24	−1.45 ± 1.04	0.23
EDIP	0.54 ± 0.25	0.53 ± 0.28	0.56 ± 0.22	0.54

^a^Data are presented as mean ± SDs unless otherwise stated.^b^ Consumption of aminosalicylic acid compounds (5-ASA) or immune modulators, i.e. azathioprine, prednisolone and methotrexate, each per se.^c^ Combination at least two classes of drugs, including ASA, immune modulator, and anti-tumor necrosis factor (TNF) agents, i.e., Remicade® infliximab and Humira® (adalimumab). *BMI* Body mass index, *DII* Dietary inflammatory index inflammatory bowel disease, *EDIP* Empirically derived inflammatory pattern, *IBD* Inflammatory bowel disease

Characteristics of participants based on tertiles of inflammatory indices are presented in Table 3, except for BMI, which was higher significantly among those in the second and third tertiles of the DII compared to the first, other characteristics did not differ significantly based on tertiles of the scores.

**Table 3:** Participants characteristics based on tertiles of dietary inflammatory scores

Variables	Inflammatory indexes			*P*-value^1^
	Tertile 1	Tertile 2	Tertile 3	
Dietary Inflammatory Index				
Number	48	49	46	–
Age, years	41 ± 9.87	37.8 ± 11.3	37.2 ± 11.0	0.19
Female, n (%)	23 (47.9)	26 (53.1)	17 (37.0)	0.28
BMI, kg/m^2^	26.8 ± 2.54	24.5 ± 2.96	23.6 ± 2.72	< 0.001
Smokers, n (%)	10 (20.8)	6 (12.2)	6 (13)	0.44
Education, n (%)				
< 14 years of formal education	4 (8.33)	9 (18.4)	13 (28.3)	0.06
Bachelor’s degree	25 (52.1)	29 (59.2)	19 (41.3)	
≥ Master’s degree	19 (39.6)	11 (22.4)	14 (30.4)	
Empirically Derived Inflammatory Pattern				
Number	48	47	48	**–**
Age, years	38.2 ± 10.3	36.9 ± 11.1	40.9 ± 10.8	0.18
Female, n (%)	23 (47.9)	25 (53.2)	18 (37.5)	0.30
BMI, kg/m^2^	24.8 ± 3.12	24.8 ± 2.69	25.4 ± 3.26	0.47
Smokers, n (%)	3 (6.2)	8 (17)	11 (22.9)	0.07
Education, n (%)				
< 14 years of formal education	15 (31.2)	5 (10.6)	6 (12.5)	0.05
Bachelor’s degree	18 (37.5)	28 (59.6)	27 (56.2)	
≥ Master’s degree	15 (31.2)	14 (29.8)	15 (31.2)	

^1^ ANOVA-test and Chi-square test were used for continuous and categorical variables, respectively

Odds ratios (OR) and 95% confidence intervals (CI) of having active disease in IBD patients, based on tertiles and median of inflammatory diet scores are shown in Table 4. No significant association was observed between the DII**®** and disease activity, either using categorization of DII by tertiles or median. Risk of having active disease was higher with higher EDIP scores, tertile or median, but none of these findings reached statistical significance.

**Table 4:** Odds ratios (95%CI) of disease activity in IBD patients based on the dietary inflammatory scores^a^

Dietary Inflammatory scores	Tertile 1	Tertile 2	Tertile 3	> Median
Dietary Inflammatory Index				
Number	48	49	46	
Median	−2.31	−1.56	0.01	−1.57
Min, Max	−3.66, −1.93	−1.92, −1.09	−1.07, 1.99	−3.67, 1.99
Univariate	1	1.33 (0.60–2.98)	0.83 (0.36–1.89)	0.78 (0.40–1.50)
Adjusted^a^	1	1.28 (0.52–3.17)	0.84 (0.32–2.17)	0.79 (0.37–1.68)
Empirically Derived Inflammatory Pattern				
Number	48	47	48	
Median	0.3	0.53	0.8	0.54
Min, Max	−0.25, 0.44	0.45, 0.66	0.67, 1.31	−0.25, 1.31
Univariate	1	1.34 (0.6–3.03)	1.40 (0.62–3.15)	1.61 (0.83–3.14)
Adjusted^a^	1	1.57 (0.65–3.81)	1.50 (0.61–3.72)	1.72 (0.83–3.59)

^a^Adjusted for total energy intake (continuous), type of disease (CD /UC) and drug consumption (no drugs, single drug, and multiple drugs)

## Discussion

In this study, we investigated associations between the inflammatory properties of diets and disease activity in IBD patients. No significant association was observed between either DII**®** or EDIP and disease activity in IBD patients.

Inflammation exacerbates symptoms of IBD [1]. There is some evidence suggesting that inflammatory state can be modulated by food parameters [29]. Therefore, dietary intakes may affect progression and severity of IBD via their effects on inflammation [30]. Lacking data on inflammatory markers, we sought to examine the effect of the inflammatory potential of an individual’s diet by two dietary scores of DII and EDIP calculated from a Persian FFQ [15, 16]. The DII, mainly nutrient-based index, was developed by reviewing all data documented by studies on the association of food items, nutrients, and non-nutrient components with six inflammatory markers [15]. The EDIP, a food-group based index, was developed based on a posteriori pattern in an American population, using reduced rank regression [16].

Patients with IBD tend to change their dietary intakes because they believe that certain foods exacerbate their clinical symptom [13]. Despite the belief, limited evidence is available on associations of dietary components and disease activity in these patients. In a prospective study, meat, particularly red meat and processed meat, alcoholic beverages, and protein intakes were associated with increased likelihood of relapse in UC patients [31]. A cross-sectional study of 103 adults with IBD reported a 79% lower risk of active disease in the highest quartiles of legumes and potato intakes versus the lowest [32]; these studies could not find any significant associations between cereals, fruits and vegetables, dairy products, fish [31, 32], carbohydrate, fat, fiber intakes and disease activity [31]. Considering the anti-inflammatory properties of fish and n-3 fatty acids, the effects of n-3 fatty acids on disease activity have been examined in clinical trials but no beneficial effects were reported [12, 33].

Despite point estimates trending in the expected direction, findings of our study were not consistent with the hypothesis that diet-induced inflammation increases disease activity in IBD patients. The combination of its small sample size and potential mask by complex factors involving the pathogenesis of IBD may have obscured a relationship. Slight variations in the ranges of these dietary indices across participants may also result in these null findings. In the current study, 83% of the population consumed the anti-inflammatory diet based on the DII, suggesting that participants of this study may have relatively healthy dietary intakes. In addition, it is probable that inflammatory indices modulate inflammation in the patients but could not affect the clinical course, an observation that is reported for n-3 supplementation. Clinical trials suggest that n-3 fatty acid supplementation in IBD patients reduces inflammation and lessens the need for steroid therapy but does not maintain remission [34].

The association between inflammatory dietary scores and IBD has been investigated in one other study, also conducted in Iran, in which DII was computed using 27 food parameters, and results showed that greater adherence to the pro-inflammatory diet was associated with increased risk of UC; a comparison of DII between cases and controls clearly shows that healthy controls had higher adherence to anti-inflammatory diet than patients with UC [24].

To the best our knowledge, this is the first investigation of association between dietary inflammatory indices and disease activity in IBD patients. Using two dietary inflammatory scores with different concept and design is the main strength of this study; some limitations, however, also should be noted. First, the cross-sectional design of the study does not allow for control of reverse causation; i.e., changes in dietary intakes in active patients are possible. Second, these two indices had not been validated using inflammatory markers in the general Iranian population. Previous studies using different inflammatory markers showed validity of scores on predicting the inflammatory markers [17, 18, 21]. In Iran, a case-control study on gastric cancer showed the validity of the DII using 4 inflammatory markers [35] but their validity in general population of Iran has not been investigated, although it has been suggested that these scores can be adapted in different populations [15, 16]. Third, because of unavailability of information on intakes of alcohol, ginger, saffron, turmeric, thyme/oregano, rosemary, and eugenol only 38 out of 45 food parameters were used to calculate the DII score. In addition, drinking low-energy beverages, beer, and wine were not included to construct EDIP; however, excluding these items does not seem to change the inflammatory scores because of their low consumption in these populations. Fourth, FFQs are well known to be biased by a number of factors [36, 37]. This would be more of a problem in a study in which people are aware of their diagnosis, which is related to diet. This cohort of patients may adjust their diet following the IBD diagnosis and knowledge of the effect of diet on the gut, thus may introduce bias into the measure. Fifth, this study is limited by its small sample size; the fact that results were trending in the right direction highlights this concern; and lastly, CDAI and Mayo scores are the most frequently used tools to assess disease activity indirectly in patients with IBD but they may not be as accurate as direct methods of assessing intestinal inflammatory activity. Although direct methods including histopathological and endoscopical examinations provide accurate evidence of intestinal inflammatory activity, they are time consuming, invasive, and expensive, which limit their applicability in routine use in clinical settings [38].

## Conclusions

Overall, in this cohort of IBD patients, the inflammatory potential of diet assessed by the DII and EDIP was not associated with severity of disease. Identification of food components in relation to disease activity in patients with IBD can lead to better management of the disease. In this regard, the hypothesis that diet induced inflammation can affect disease activity in patients with IBD deserves further investigation.

## Data Availability

Data that support the findings of this study are available from the corresponding author upon reasonable request.

## Abbreviations

5-ASA: AminosalicylicAcid
APC: Antigen-Presenting Cells
CD: Crohn’sDisease
CDAI: Crohn’sDisease Activity Index
CHI: Connecting Health Innovations
CI: Confidence Intervals
CRP: C-Reactive Protein
DII®: Dietary Inflammatory Index
EDIP: Empirically Derived Inflammatory Pattern
FFQ: Food Frequency Questionnaire
GI: Gastrointestinal
IBD: Inflammatory bowel disease
IL-1β: Interleukin-1β
IL-6: Interleukin-6
OR: Odds Ratios
Thcells: T helper Cells
TNF-alpha: Tumor Necrosis Factor-alpha
UC: Ulcerative Colitis
USDA: United States Department of Agriculture
